# TG2-gluten complexes as antigens for gluten-specific and transglutaminase-2 specific B cells in celiac disease

**DOI:** 10.1371/journal.pone.0259082

**Published:** 2021-11-03

**Authors:** Christian B. Lindstad, Alisa E. Dewan, Jorunn Stamnaes, Ludvig M. Sollid, M. Fleur du Pré

**Affiliations:** 1 K.G. Jebsen Coeliac Disease Research Centre, University of Oslo, Oslo, Norway; 2 Department of Immunology, University of Oslo, Oslo, Norway; 3 Department of Immunology, Oslo University Hospital, Oslo, Norway; Xavier Bichat Medical School, Université Paris Diderot, FRANCE

## Abstract

A hallmark of celiac disease is the gluten-dependent production of antibodies specific for deamidated gluten peptides (DGP) and the enzyme transglutaminase 2 (TG2). Both types of antibodies are believed to result from B cells receiving help from gluten-specific CD4^+^ T cells and differentiating into antibody-producing plasma cells. We have here studied the collaboration between DGP- and TG2-specific B cells with gluten-specific CD4^+^ T cells using transgenic mice expressing celiac patient-derived T-cell and B-cell receptors, as well as between B-cell transfectants and patient-derived gluten-specific T-cell clones. We show that multivalent TG2-gluten complexes are efficient antigens for both TG2-specific and DGP-specific B cells and allow both types of B cells to receive help from gluten-specific T cells of many different specificities.

## Introduction

Celiac disease is an autoimmune enteropathy driven by exposure to dietary wheat gluten (gliadin/glutenin) proteins and related proteins of barley and rye [1]. Patients have a CD4^+^ T-cell response towards post-translationally modified (deamidated) gluten peptides that selectively bind to the disease-predisposing HLA molecules HLA-DQ2.5, HLA-DQ8 or HLA-DQ2.2 [2]. In addition, both deamidated gluten peptides (DGP) and the self-protein transglutaminase 2 (TG2) become the targets of the B-cell response in celiac disease.

Both anti-DGP and anti-TG2 antibodies are exquisite diagnostic markers for celiac disease suggesting a role in pathogenesis [3]. It is questionable though whether immunoglobulins play a role as circulating effector molecules. Rather a role as the antigen receptor of B cells is more likely [4]. In this setting, efficient collaboration between B cells and T cells is essential, and B cells will be the major antigen-presenting cell type driving the inflammatory T-cell response in celiac disease [5]. In accordance with this notion it was found that anti-DGP antibodies recognize epitopes that often overlap with or are in close proximity to known gluten T-cell epitopes [6], suggesting that the gluten-reactive B-cell repertoire is ideally selected to receive help from gluten-specific T cells. The production of anti-TG2 autoantibodies is believed to be the result of the collaboration between TG2-specific B cells and gluten-specific CD4^+^ T cells following the uptake of covalent TG2-gluten complexes [7, 8]. Covalently linked complexes of TG2 and gluten can be formed in two ways; either by gluten transiently bound to TG2s active site through a labile thioester bond, or by crosslinking of gluten peptides to lysine residues in TG2 through a stable isopeptide bond [9].

We have previously shown that crosslinked TG2-gluten complexes can indeed facilitate the interaction between TG2-specific B cells and gluten-specific T cells *in vitro* [10]. Another important finding is that TG2 is an excellent substrate for itself and catalyzes the formation of TG2-TG2-gluten multimers. These TG2-TG2-gluten multimers are superior to monomers in activating TG2-specific B cells and their uptake leads to efficient antigen presentation to gluten-specific T cells [11]. Here we extend our previous findings by showing that also naive antigen-specific T and B cells isolated from T-cell receptor (TCR) and B-cell receptor (BCR) transgenic mice efficiently collaborate to TG2-gluten complexes *in vitro* and *in vivo*, and that the increased activation by multimeric complexes could not be explained by increased incorporation of gluten peptides. Furthermore, we show that TG2-gluten complexes are also preferred antigens for DGP-specific B cells. The study provides further insight into disease-driving adaptive immune processes in celiac disease.

## Methods

### Mice

Immunoglobulin heavy chain (H) and immunoglobulin light chain (L) double-KI-mice express the 679-14-E06 (from here denoted 14E06) B cell receptor from a single TG2-reactive gut plasma cell [12]. *Tgm2*^-/-^ mice [13] mice were kindly provided by Prof. G. Melino. HLA-DQ2.5 tg [14], HLA-DQ2.5 KI [15], TCR-glia-α2 tg [12] and TCR-glia-ω2 tg mice [16] are described previously. HLA-DQ2.5 tg or heterozygous targeted HLA-DQ2.5 KI mice express both HLA-DQ2.5 and H2-IA and are assumed to be functionally similar [15]. HLA-DQ2.5 tg or heterozygous HLA-DQ2.5 KI mice were crossed to 14E06 double KI-mice to obtain DQ2.5.14E06 mice. For in vitro assays, DQ2.5.14E06 mice were crossed with *Tgm2*^-/-^ mice to obtain *Tgm2*^-/-^ DQ2.5.14E06 mice. TCR-glia-α2 tg and TCR-glia-ω2 tg mice were crossed with HLA-DQ2.5 tg or heterozygous HLA-DQ2.5 KI mice to obtain DQ2.5.TCR-glia-α2 and DQ2.5.TCR-glia-ω2 mice. Mice were bred and kept in individually ventilated cages (GM 500, Techniplast) under specific pathogen-free conditions at the Department of Comparative Medicine, Oslo University Hospital, Rikshospitalet (Oslo, Norway). All mice are on C57Bl/6 background. Mice were kept on gluten-free mouse chow (Research Diets D11112201). All experiments were approved by the Norwegian Food Safety Authority (Mattilsynet), protocol numbers 10897, 12414 and 15311. All efforts were made to minimize animal suffering.

### Antigens

The following synthetic peptides were purchased from Genscript or GL Biochem: native 33mer α-gliadin: LQLQPFPQPQLPYPQPQLPYPQPQLPYPQPQPF, deamidated 33mer α-gliadin: LQLQPFPQPELPYPQPELPYPQPELPYPQPQPF, native ω-gliadin: QPQQPFPQQPQQPQQPFPQPQQPFPWQPQQPFPQ, deamidated 34mer ω-gliadin: QPQQPFPEQPQQPEQPFPQPEQPFPWQPEQPFPQ. Mouse and human TG2 and mouse TG2-α33merEEE fusion protein were produced in expresSF+ Serum Free Insect Cell Line cells (Protein Sciences) as previously described [12]. TG2-gluten complexes were generated by incubating TG2 with native gluten peptides for 30–40 min at 37°C in a Tris-buffer containing 5 mM CaCl_2_. Catalytic activity of TG2 was then inhibited by adding 5 mM iodoacetamide. Monomers and multimers were separated by size-exclusion chromatography as described [11]. SDS PAGE confirmed adequate separation of monomeric and multimeric TG2-α33merEEE fusion protein and TG2-33mer monomeric and multimeric complexes: proteins (3 μg) were run on 4–20% TGXgels (BioRad) (S1 Fig).

### *In vitro* T cell B cell collaboration assay—Mouse primary cells

Single-cell suspensions were prepared by passing tissues through a 70 μM nylon strainer (Falcon) followed by ammonium-chloride potassium lysis of erythrocytes. B cells were isolated from spleens using Dynabeads Mouse CD43 kit (Invitrogen). B-cell purity measured as percentage of cells that were B220^+^ was typically >95%. CD4^+^ T cells were isolated from spleens and lymph nodes (LNs) using EasySep Mouse CD4^+^ T cell isolation kit (StemCell Technologies). CD4^+^ T cell purity was typically > 90%. Before culture, cells were labeled with proliferation-tracking dyes Cell Trace Violet (CTV) or Cell Trace CFSE (both Thermo Fisher Scientific). For *in vitro* proliferation assays, 200 000 B cells and 40 000 CD4^+^ T cells were cultured in 96 well round-bottom plates for three days in a gassed incubator at 37°C. Assays were set in duplicates or triplicates. RPMI-1640 supplemented with 10% (vol/vol) FCS, penicillin, streptomycin, 100 μM β-mercaptoethanol, 1 mM sodium pyruvate, 0.1 mM non-essential amino acids and 10 mM hepes was used as culture medium.

### *In vivo* T cell B cell collaboration assay

On day -5, 2 x 10^6^ or 4 x 10^6^ CD4^+^ T cells isolated from DQ2.5.TCR-glia-α2 mice were injected intravenously into HLA-DQ2.5 recipient mice. The cells were primed by intraperitoneal administration of 50 μg deamidated 33mer α-gliadin or 34mer ω-gliadin peptide in 50 μl PBS emulsified with 50 μl Complete Freund’s Adjuvant (CFA, Sigma-Aldrich). On day -1, 1 x 10^7^ DQ2.5.14E06 B cells were labeled and transferred intravenously. The next day, mice were injected in both thigh muscles with in total 50 or 100 μg antigen in 50 μl PBS (25 μl in each leg). B cell proliferation in draining popliteal and inguinal LNs was analyzed on day +6.

### Flow cytometry

Mouse primary cells were resuspended in PBS 2% (vol/vol) FCS and stained with antibodies against the following molecules: B220 (RA3-6B2, BioLegend), CD4 (GK1.5; Biolegend), DQ2.5 β-chain (2.12.E11, [17]). 14E06 expression on B cells was detected with an unconjugated human anti-14E06 antibody (2G9, [18]) followed by PE-conjugated anti-human IgG1 (4E3, Acris Antibodies). Viability was assessed using 7-amino-actinomycin D (7-AAD, eBioscience).

Transfectant A20 B cells were stained with gluten peptide tetramers for 30 min on ice. Tetramers were generated by incubating biotinylated deamidated 34mer ω-gliadin (biotin-QPEQPFPEQPEQPEQPFPQPEPFPWQPEQPFPQ) or 33mer α-gliadin (biotin-GSGSGS-LQLQPFPQPELPYPQPELPYPQPELPYPQPQPF, both GL Biochem) with SA-APC (PhycoLink) at a 4:1 molar ratio for 2 hours in PBS 2% FCS at 4°C. Samples were acquired on FacsCalibur (BD) or Attune Nxt Flow Cytometer (Thermo Fisher Scientific). Data were analyzed with FlowJo software (BD).

### *In vitro* T cell B cell collaboration assay–A20 cells and human T cell clones

Murine A20 B lymphoma cells expressing HLA-DQ2.5 and the DGP-reactive 1130-3-B01 IgD BCR [19] were generated by retroviral transduction as previously described for 679-14-E06 and 693-2-F02 BCRs [10]. Gluten-specific T-cell clones derived from HLA-DQ2.5^+^ patients with celiac disease were used (epitope specificity in parentheses): TCC 430.1.57 (DQ2.5-glia-α1a), TCC 387.9 (DQ2.5-glia-α1a), TCC 436.5.3 (DQ2.5-glia-α2), TCC1383.P.A.6 (DQ2.5-glia-ω1) and TCC 737.30 (DQ2.5-glia-ω2). A20 B cells were irradiated at 75 Gy and incubated with indicated concentrations of deamidated 33mer α-gliadin or 34mer ω-gliadin peptides for 5 min on ice, then washed with cold RPMI to remove unbound antigen and cultured overnight at 75,000 cells per well. Incubation with TG2-gluten complexes was done for 1 h at room temperature, followed by washing and culturing as above. For incubation with native peptides in the presence of active TG2, A20 cells were incubated with 50 nM hTG2 and indicated concentrations of native 33mer α-gliadin or 34mer ω-gliadin peptides in RPMI supplemented with 2 mM CaCl_2_ for 5 min at 37°C, washed and cultured as above. The next day, 50,000 gliadin-specific T cells were added to each well. T-cell proliferation was measured by the uptake of [^3^H] thymidine (1 μCi/well), which was added to the wells 16 h before harvesting. Cells were harvested 72 h after T cells were added onto glass fiber paper with an automated harvester (Mach III, Tomtec) and [^3^H] thymidine incorporation was measured by liquid scintillation counting (Wallac MicroBeta TriLux 1450 or MicroBeta 2450, Perkin Elmer). The patients from whom T-cell clones and TCR- and BCR-sequences were derived had signed written informed consents being enrolled in a project with ethical approval from the Regional Committee for Medical and Health Research Ethics–South East Norway (project 6544), project leader Knut E.A. Lundin).

### Data analysis

Data was visualized with Graphpad Prism 8.0.1 (GraphPad Software).

## Results

### A TG2-gluten fusion protein elicits specific collaboration between TG2-specific B cells and gluten-specific T cells *in vitro*

**Fig 1:**
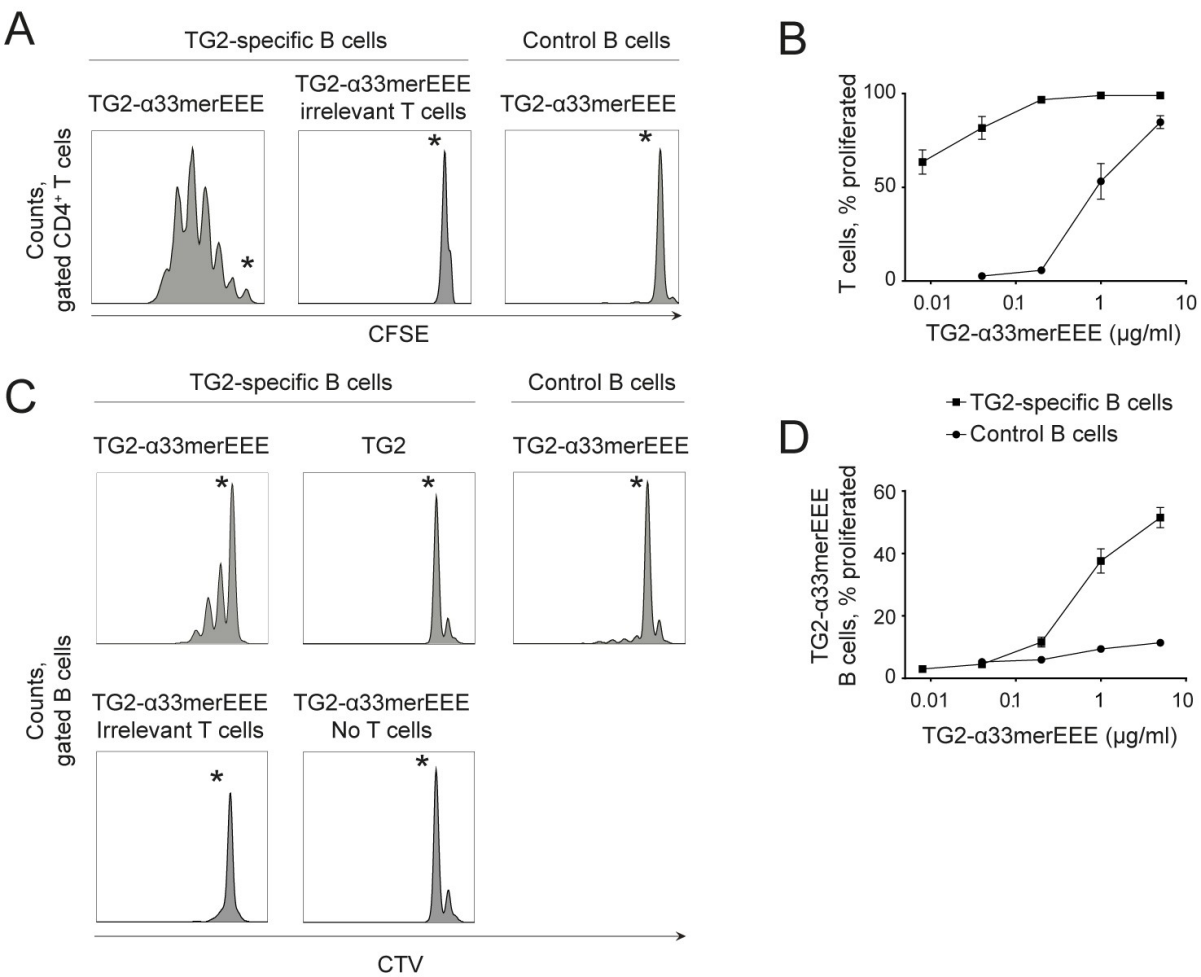
TG2-specific B cells and gluten-specific CD4^+^ T cells interact in vitro. B cells isolated from *Tgm2*^*-/-*^ DQ2.5.14E06 or HLA-DQ2.5 mice, and CD4^+^ T cells isolated from DQ2.5.TCR-glia-α2 or DQ2.5.TCR-glia-ω2 mice were labeled with different proliferation-tracking dyes and co-cultured in vitro for three days stimulated with indicated antigens. (A) Representative histogram plots gated on CD4^+^ T cells showing proliferation in response to TG2-α33merEEE fusion protein at 0,2 μg/mL. (B) Dose response curve of T-cell proliferation to TG2-α33merEEE fusion protein. (C) Representative histogram plots gated on B220^+^ B cells showing proliferation in response to TG2-α33merEEE fusion or TG2 at 5μg/mL. (D) Dose response curve of B-cell proliferation to titrations of TG2-α33merEEE fusion protein. Asterisk (*) indicates the undivided peak. Graphs report proliferation as % proliferated CD4^+^ T or B220^+^ B cells. Points and bars represent mean +/- SEM. Data is representative of three independent experiments set in duplicates. CFSE: Cell Trace Carboxyfluorescein Succinimidyl Ester CTV: Cell Trace Violet.

We first determined that naive TG2-specific B cells and naive gluten-specific T cells are able to interact *in vitro*. Naive TG2-specific B cells were isolated from immunoglobulin knock-in (KI) mice that express a celiac patient-derived anti-TG2 B-cell receptor (14E06) in addition to HLA-DQ2.5 [12]. The DQ2.5.14E06 KI mice were also *Tgm2-*deficient to avoid release of TG2 antigen during tissue processing. TG2-specific B cells were cultured with gluten-specific CD4^+^ T cells recognizing the DQ2.5-glia-α2 epitope isolated from HLA-DQ2.5-positive gluten-specific T-cell receptor transgenic mice [12]. A recombinant fusion protein of mouse TG2 and the deamidated 33mer α-gliadin peptide harboring the deamidated T-cell epitope DQ2.5-glia-α2 (from here denoted TG2-α33merEEE fusion protein) was used as antigen. When TG2-specific B cells were used, strong T-cell proliferation occurred at antigen concentrations as low as 0.01 μg/mL, reflecting specific uptake of TG2-α33merEEE through the TG2-specific B cell receptor and presentation of deamidated gluten peptide to T cells (Fig 1A and 1B). No proliferation of irrelevant DQ2.5-glia-ω2-reactive CD4^+^ T cells was observed (Fig 1A). In turn, activated gluten-specific CD4^+^ T cells provided help and induced proliferation of TG2-specific B cells (Fig 1C and 1D). B-cell proliferation was greatly reduced when cognate T-cell help was unavailable (Fig 1C). Taken together, these data demonstrate that naive TG2-specific B cells and gluten-specific T cells are able to collaborate in an antigen specific manner *in vitro*.

### TG2-gluten multimers are superior to TG2-gluten monomers at inducing antigen-specific proliferation of T and B cells

**Fig 2:**
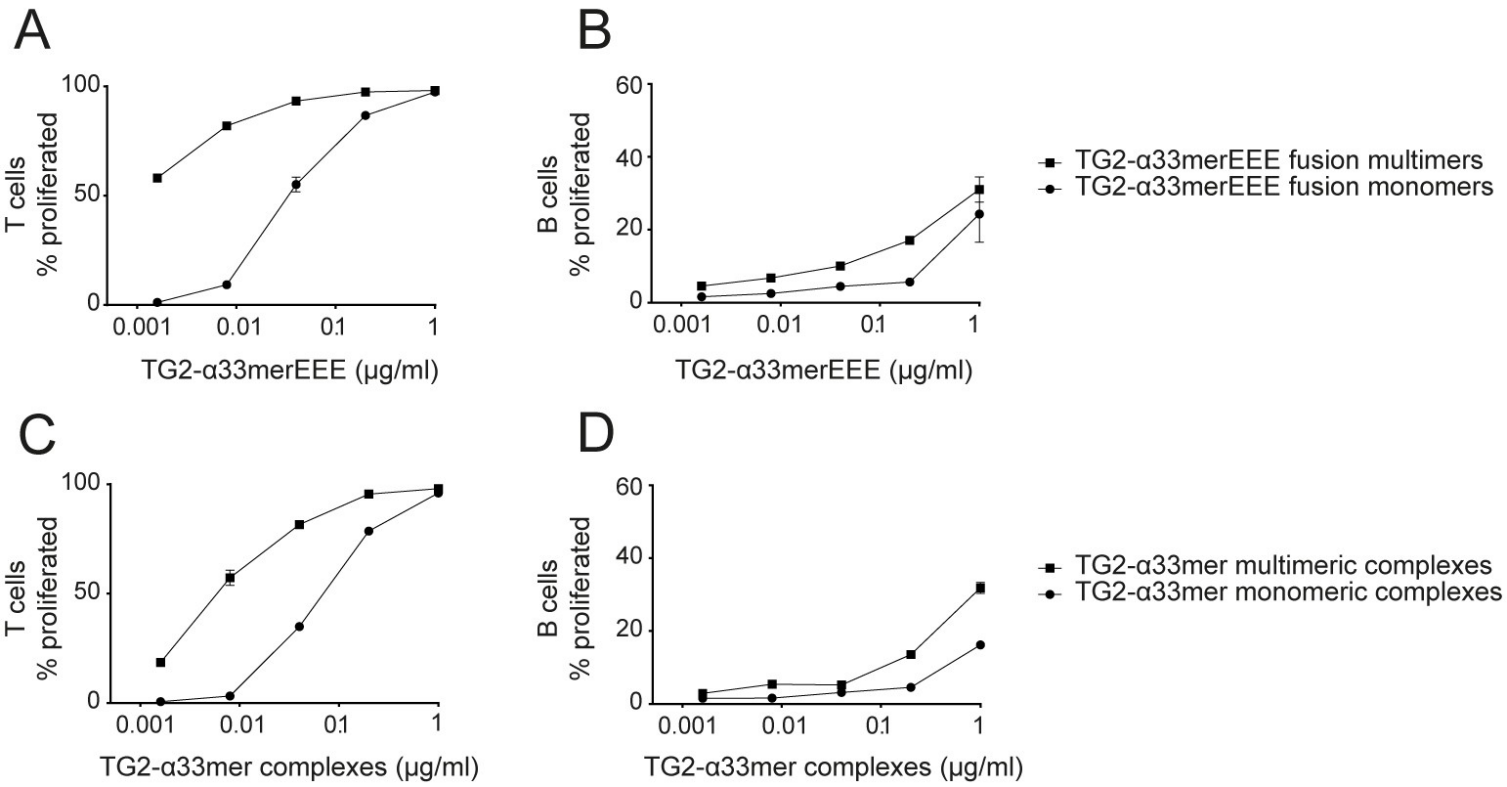
TG2-gluten multimers are superior to monomers at activating gluten-specific T cells and TG2-specific B cells. B cells isolated from *Tgm2*^*-/-*^ DQ2.5.14E06 mice and CD4^+^ T cells isolated from DQ2.5.TCR-glia-α2 mice were labeled with different proliferation-tracking dyes and co-cultured in vitro for three days stimulated with indicated antigens. (A,B) Dose response curve of proliferative response to TG2-α33merEEE fusion monomers and multimers for CD4^+^ T cells (A) and B cells (B). (C,D) Dose response curve of proliferative response to TG2-α33mer monomers and multimers for CD4^+^ T cells (C) and B cells (D). Graphs report proliferation as % proliferated when gated on dye-labeled CD4^+^ or B220^+^ cells. Points and bars represent mean +/- SEM. Data is representative of three independent experiments set in duplicates.

Multivalent antigens are capable of crosslinking B-cell receptors on the cell surface, therefore TG2-gluten multimers are expected to be superior to monomers in activating naive antigen-specific B cells. To generate multivalent antigen, we incubated the TG2-α33merEEE fusion protein in a calcium-containing buffer allowing for self-crosslinking, followed by separation into monomers and multimers. Importantly, the TG2:gluten ratio in these proteins is 1:1. B cells from *Tgm2*^*-/-*^ DQ2.5.14E06 mice were cultured *in vitro* with CD4^+^ T cells from DQ2.5.TCR-glia-α2 mice and TG2-α33merEEE monomers and multimers. The multimeric antigen was clearly superior to the monomer in inducing proliferation of gluten-specific T cells (Fig 2A). We also observed increased proliferation of TG2-specific B cells in response to the multimeric antigen (Fig 2B). We next moved on to more physiologically relevant TG2-α33mer complexes to confirm our previous observations using transfected A20 B cells [11] with isolated naive antigen-specific B and T cells from mice. TG2-α33mer complexes were generated by incubating mouse TG2 with synthetic native 33mer α-gliadin peptide in a calcium-containing buffer, before separation into monomers and multimers. These complexes also induced robust proliferation of gluten-specific T cells and TG2-specific B cells. Multimers were highly superior to monomers on the T-cell side and moderately superior on the B-cell side (Fig 2C and 2D). Thus, the effect of multivalency is seen both when the TG2-gluten ratio is 1:1 and when the TG2-gluten ratio is unknown. These results demonstrate that TG2-gluten complexes elicit antigen-specific activation of both gluten-specific T cells and TG2-specific B cells, and that multivalent TG2-gluten complexes are more potent than monovalent complexes *in vitro*.

### TG2-specific B cells and gluten-specific CD4^+^ T cells collaborate *in vivo* in response to pre-formed TG2-gluten complexes

**Fig 3:**
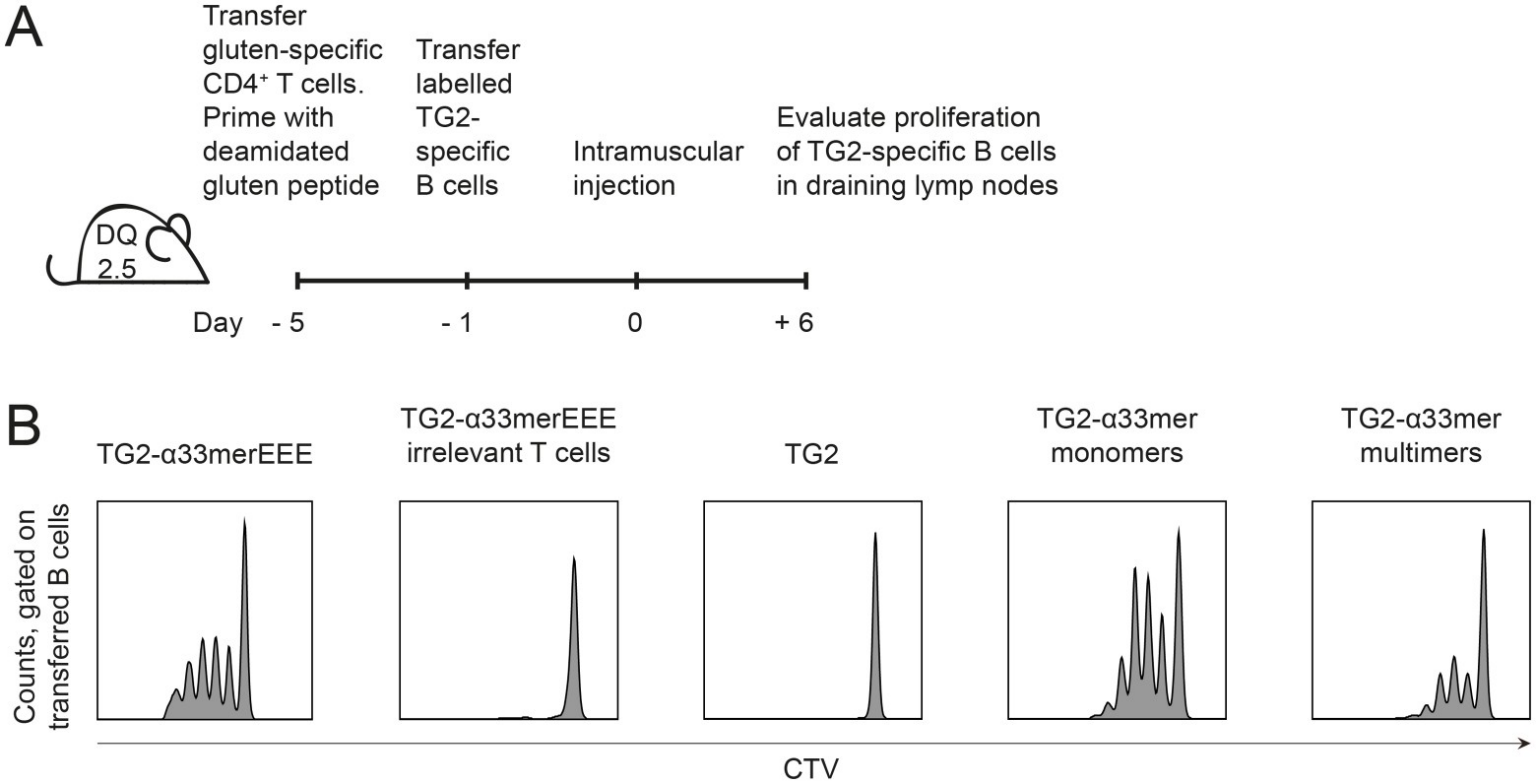
TG2-specific B cells expand in vivo upon help from gluten-specific CD4^+^ T cells. (A) Experimental setup: CD4^+^ T cells isolated from DQ2.5.TCR-glia-α2 or DQ2.5.TCR-glia-ω2 mice were administered intravenously to HLA-DQ2.5 expressing recipient mice and primed with cognate deamidated 33mer α-gliadin or cognate deamidated 34mer ω-gliadin peptides emulsified in CFA. TG2-specific B cells were isolated from DQ2.5.14E06 mice, labeled with CTV and administered intravenously 4 days after T-cell priming. Antigen was administered intramuscularly the following day. B cell proliferation was assayed by flow cytometry 6 days after immunization. (B) Representative histogram plots gated on B220^+^ 14E06^+^ cells show B cell proliferation in draining popliteal and inguinal LNs. The panel shows response to TG2-α33merEEE fusion protein, TG2, and to pre-formed monomeric and multimeric TG2-α33mer complexes. Data are representative of in total n = 6 (TG2-α33merEEE fusion protein, TG2) or n = 4 (TG2-α33mer monomers, multimers) from at least 3 independent experiments). CTV: Cell Trace Violet.

Next, we examined whether TG2-specific B cells and gluten-specific T cells can collaborate *in vivo* upon administration of TG2-gluten complexes using an adoptive transfer model (Fig 3A). HLA-DQ2.5 mice received naive gluten-specific CD4^+^ T cells from DQ2.5.TCR-glia-α2 or DQ2.5.TCR-glia-ω2 mice. The T cells were subsequently primed by injection of deamidated gluten peptides emulsified in CFA. After 4 days, DQ2.5.14E06 B cells were transferred and mice were immunized intramuscularly with TG2-α33merEEE fusion protein or TG2. As observed *in vitro*, TG2-specific B cells in draining LNs proliferated to TG2-α33merEEE fusion only when cognate T-cell help was provided (Fig 3B). We then repeated the experiment using TG2-α33mer complexes. TG2-α33mer complexes induced proliferation of DQ2.5.14E06 B cells, while TG2 did not (Fig 3B). In contrast to our *in vitro* observations, no significant difference was observed between monomers and multimers. Taken together, these observations confirm that TG2-gluten complexes can elicit antigen-specific collaboration between TG2-specific B cells and gluten-specific T cells *in vivo*.

### DGP-specific B cells efficiently bind deamidated gliadin peptides

We next hypothesized whether TG2-gluten complexes could also serve as antigens for DGP-specific B cells. DGP-specific B cells recognize deamidated gluten epitopes. The sequence QPEQPF has been described as the immunodominant epitope for DGP-specific B cells and is often found in many copies in gluten proteins harboring known T-cell epitopes [6, 20]. We have previously shown that transduced lymphoma B cells expressing a B-cell receptor recognizing DGP efficiently bind a 34mer ω-gliadin peptide, containing three copies of the B-cell core epitope, and stimulated T cells more efficiently than B cells transduced with an irrelevant BCR [21]. Since many gluten-specific plasma cells were shown to cross-react with different gliadin peptides, we have now generated A20 B lymphoma cells expressing HLA-DQ2.5 as well as a gluten-specific BCR (1130-3-B01, from here denoted 3B01) recognizing both the B cell core epitope (QPEQPF) epitope and the deamidated 33-mer [19]. The 33mer peptide from α-gliadin is often considered the most immunodominant gluten peptide and harbors the DQ2.5-glia-α1 and DQ2.5-glia-α2 T-cell epitopes, but does not contain the QPEQPF sequence.

**Fig 4:**
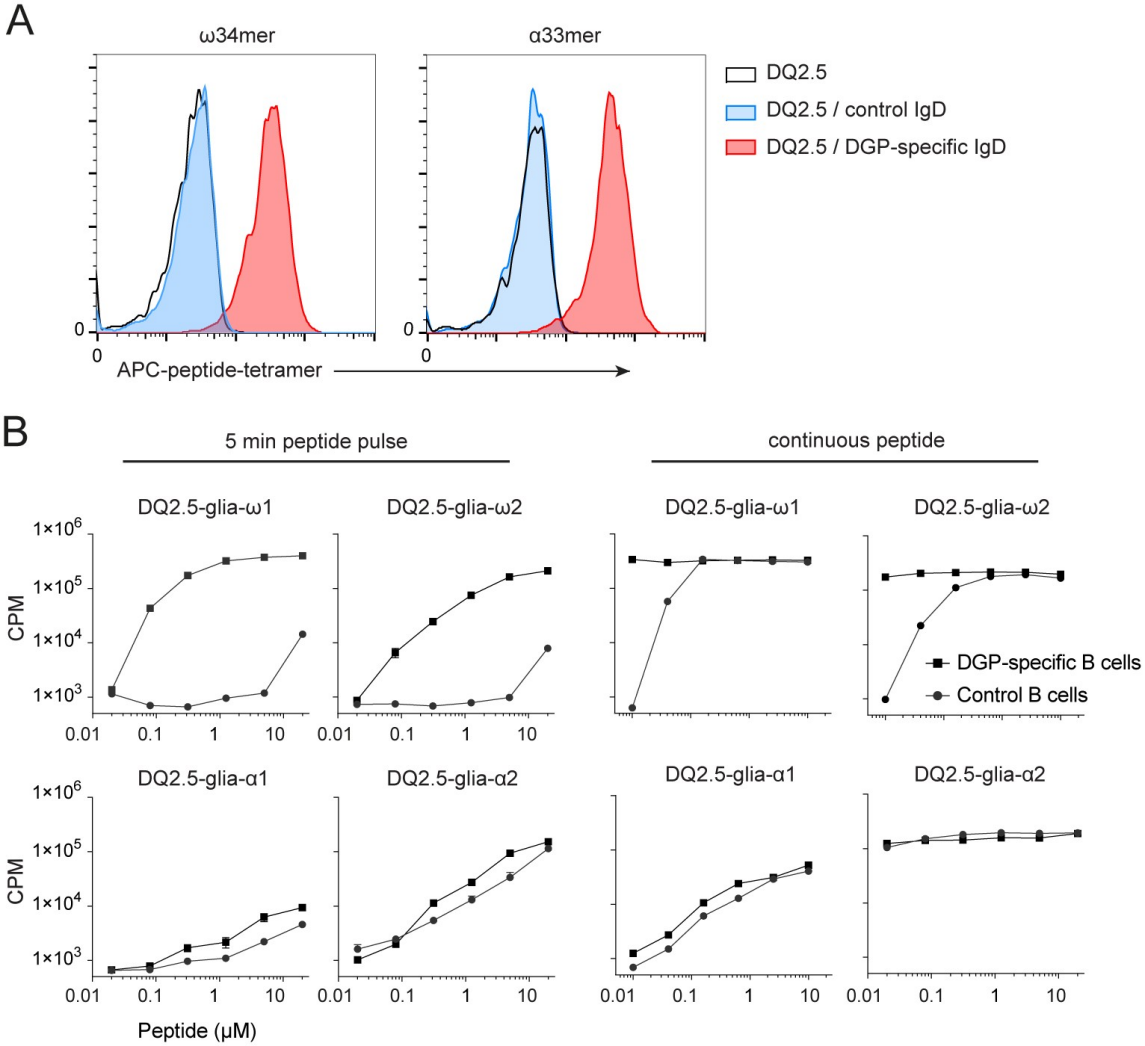
DGP-specific B cells efficiently bind deamidated gliadin peptides. (A) Binding of deamidated ω34mer and α33mer gluten peptide by A20 transfectant B cells expressing either HLA-DQ2.5 alone (black line, no fill), HLA-DQ2.5 and an irrelevant IgD BCR (693-2-F02; blue) or HLA-DQ2.5 and the gluten-specific IgD BCR 1130-3-B01 (red). Cells were stained with biotinylated deamidated ω34mer or α33mer that was multimerized with APC-conjugated streptavidin. (B). Presentation of deamidated gluten peptides by irradiated transfectant A20 B cells expressing HLA-DQ2.5 and the DGP-specific 3B01 IgD BCR or the 2F02 control BCR. A20 B cells were either pulsed with indicated concentrations of deamidated ω34mer or α33mer peptide for 5 min on ice, or peptides were present continuously during the assay. A20 B cells were cultured overnight at 37°C before gluten-specific T cells specific for the DQ2.5-glia-ω1 (TCC 1383.P.A.6), DQ2.5-glia-ω2 (TCC 737.30), DQ2.5-glia-α1 (TCC 430.1.57) and DQ2.5-glia-α2 (TCC 436.5.3) epitopes were added. T-cell proliferation was assessed by [^3^H] thymidine incorporation 72h later. Symbols represent mean counts per minute (CPM) +/- SEM of culture duplicates or triplicates and are representative of at least 3 independent experiments.

The A20 B cells expressing the 3B01 IgD BCR efficiently bound both biotinylated deamidated 34-mer ω-gliadin epitope, as well as biotinylated deamidated 33-mer α-gliadin peptide that were multimerized with APC-conjugated streptavidin (Fig 4A). When the DGP-reactive A20 B cells were pulsed with the deamidated 34-mer ω-gliadin peptide and cocultured with T-cell clones specific for the DQ2.5-glia-ω1 or DQ2.5-glia-ω2 epitopes, there was a clear effect of BCR-mediated uptake of the ω-34mer peptide, resulting in increased proliferation of DQ2.5-glia-ω1 and DQ2.5-glia-ω2-restricted T-cell clones (Fig 4B). In contrast, we did not observe BCR recognition of the α-33mer peptide during this short antigen pulse (Fig 4B), which could reflect a lower avidity of the BCR for α-gliadin peptides.

### TG2-gluten complexes serve as antigens for both TG2 and DGP-specific B cells in their interaction with T cells

**Fig 5:**
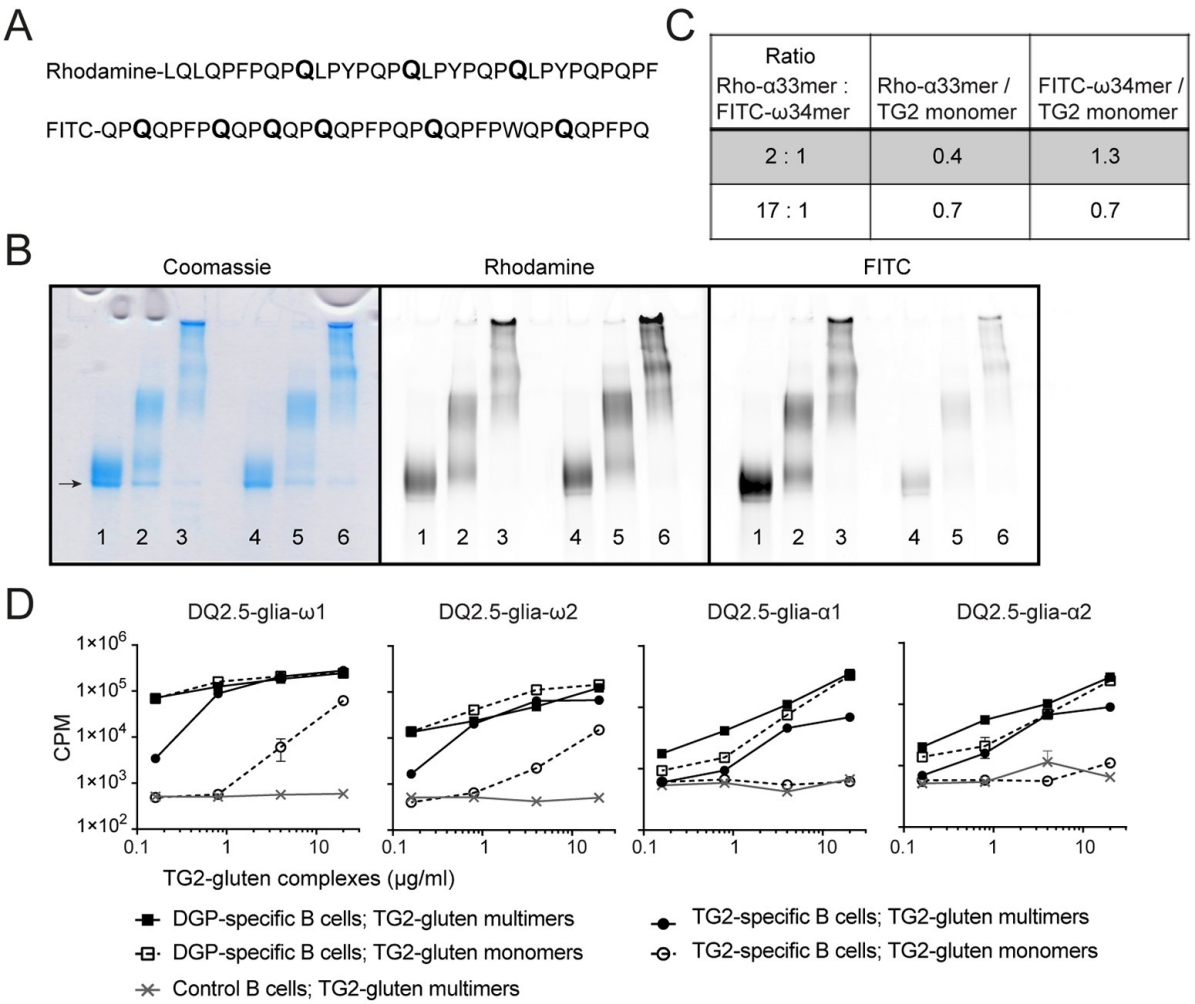
TG2-gluten complexes are efficient antigens for both DGP-specific and TG2-specific B cells and allow for help of T cells with many different specificities. (A) Amino acid sequences of the rhodamine-labeled α33mer and the FITC-labeled ω34mer peptides. Glutamine residues expected to be targeted by TG2 are enlarged and given in bold. (B) Gradient SDS-PAGE (4–20%) showing incorporation of FITC-conjugated ω-34mer and Rhodamine-conjugated α33mer into both TG2 monomers, dimers and multimers. The gel shows TG2-gluten complexes separated into monomers (lanes 1, 4), dimers/ trimers (lanes 2, 5) and multimers (lanes 3, 6) made using a 2:1 (lanes 1–3) or 17:1 (lanes 4–6) molar ratio of α33mer: ω34mer peptide. Arrow indicates monomeric TG2 (78 kDa). (C) The ω34mer peptide is crosslinked to TG2 to a higher degree than the α33mer peptide. Protein concentration and fluorescent labels were quantified using a Nanodrop UV-Vis spectrophotometer, and the number of fluorescent peptides per TG2 molecule were calculated. The table shows the average values of monomers, dimers/trimers and multimers of one representative experiment. (D) Irradiated A20 B cells expressing HLA-DQ2.5 and a TG2-specific BCR (14E06), gluten-specific BCR (3B01) or non-specific BCR (2F02) were incubated with complexes of TG2 and FITC-labeled native ω34mer gliadin peptide and rhodamine B-labeled α33mer gliadin peptide, and their ability to present deamidated peptides to TCC specific for the DQ2.5-glia-ω1 (TCC1383.P.A.6), DQ2.5-glia-ω2 (TCC737.30), DQ2.5-glia-α1 epitope (TCC430.1.57) or DQ2.5-glia-α2 epitope (TCC436.5.3) was assessed by [^3^H] thymidine incorporation. Symbols represent mean counts per minute (CPM) +/- SEM of culture triplicates. Data are representative of 3 independent experiments.

**Fig 6:**
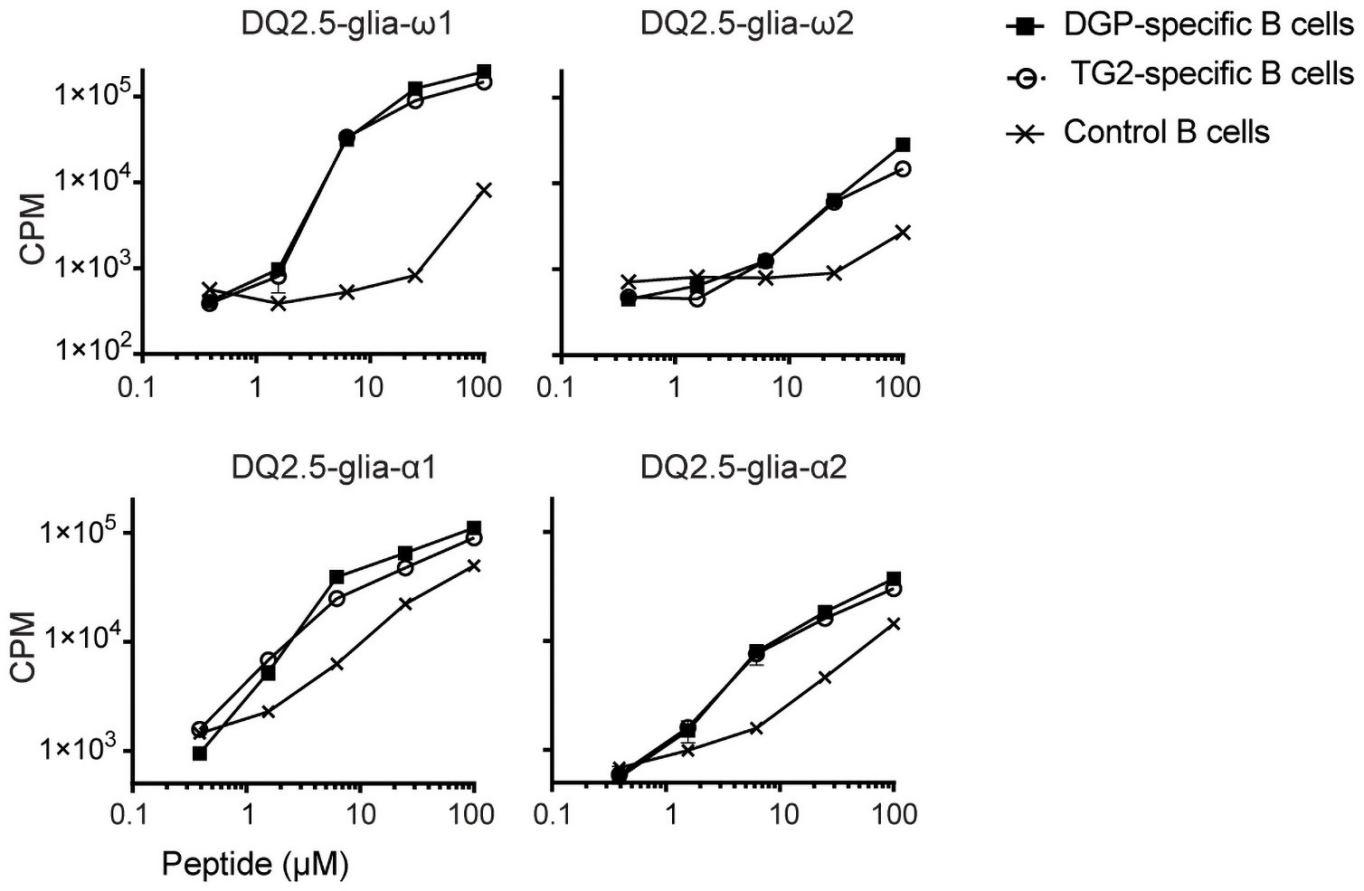
Activation of gluten-specific T cells following pulsing of A20 B cells with active TG2 and native gluten peptides. (A) Irradiated A20 cells expressing HLA-DQ2.5 and a TG2-specific BCR (14E06), gluten-specific BCR (3B01) or non-specific BCR (2F02) were incubated with different concentrations of native ω34mer or native α33mer gliadin peptide in the presence of 50 nM TG2 and 2 mM CaCl_2_ for 5 min at 37°C, then washed and cultured at 37°C. The next day, gluten-specific TCC specific for the DQ2.5-glia-ω1 (TCC1383.P.A.6), DQ2.5-glia-ω2 (TCC737.30), DQ2.5-glia-α1 epitope (TCC387.9) or DQ2.5-glia-α2 epitope (TCC436.5.3) were added. T-cell proliferation was assessed after 72h by [^3^H] thymidine incorporation. Symbols represent mean counts per minute (CPM) +/- SEM of culture duplicates and are representative of 2–3 independent experiments.

As TG2-gluten complexes allow for the cognate interaction between TG2-specific B cells and gluten-specific T cells, we next addressed if uptake of these complexes by DGP-specific B cells also results in activation of gluten-specific T cells. We incubated TG2 with a mix of fluorescently-labeled native α33mer and ω34mer gluten peptides in the presence of Ca^2+^. The native α33mer contains three glutamine residues (Q) that are targeted by TG2, whereas the native ω34mer contain six Q that can be targeted for deamidation / crosslinking (Fig 5A). To make up for this difference, the α33mer was added in double molar amounts. Gradient SDS page of size fractioned TG2-gluten complexes followed by fluorescence scanning confirmed cross-linking of both peptides to TG2 (Fig 5B). We then used a NanoDrop spectrometer to estimate the degree of incorporation of the two peptides, and found that, when using a 2:1 ratio of native α33mer: ω34mer, the ω34mer peptide was approximately 3-fold more readily crosslinked to TG2 then the α33-mer peptide (Fig 5C). Increasing the molar ratio of α33mer: ω34mer to 17:1 resulted in approximately equal incorporation of both peptides to TG2 (Fig 5B and 5C). Subsequently, we offered size-fractioned monomers of crosslinked TG2-gluten complexes (2:1 ratio of α33mer: ω34mer) to irradiated transfected A20 cells and incubated for 1 h followed by washing off unbound antigen and overnight incubation at 37°C. The next day, T-cell clones specific for the DQ2.5-glia-α1, DQ2.5-glia-α2, DQ2.5-glia-ω1 and DQ2.5-glia-ω2 T-cell epitopes were added. T-cell proliferation was assessed by [^3^H] Thymidine incorporation. We show that A20 cells expressing a DGP-specific BCR, like those expressing TG2-specific BCR, take up and internalize the TG2-α33mer-ω3mer complexes and induce the proliferation of T cells with different specificities (Fig 5D). Especially for the ω-gliadin reactive T-cell clones, we could detect strong T-cell proliferation as low as 0.1 μg/ml (equaling ~1.6 nM ω34mer peptide). When comparing multimeric and monomeric complexes we observed, in line with previous observations, a clear difference in inducing T-cell proliferation when TG2-specific A20 B cells were used as antigen presenting cells. However, when monomeric and multimeric complexes were offered to DGP-specific A20 B cells no difference was observed (Fig 6D), indicating that monomeric TG2-gluten complexes decorated with multiple gluten peptides can serve as multivalent antigens for DGP-specific B cells. In summary, our data indicate that, by incorporating many different gluten peptides, TG2 could work as a scaffold that would not only allow TG2-specific B cells but also DGP-specific B cells to receive help from T cells with different gluten specificities.

A key question remains whether cross-linked TG2-gluten complexes can be formed *in vivo*, and where active TG2 encounters gluten. It may very well be that *in vivo*, transient enzyme-substrate intermediate complexes rather than the stable crosslinked complexes we have so far used in this study are the more relevant antigen for facilitating the interaction between TG2-specific B cells and gluten-specific T cells. The fact that these enzyme-substrate intermediate complexes rely on a labile thioester bond makes it challenging to design experiments addressing this question. In an attempt to determine whether the enzyme-substrate intermediate also allows for the collaboration of DGP- and TG2-specific B cells *in vitro*, we chose to incubate transfected A20 B cells with enzymatically active TG2 and native gluten peptides during a short (5 min) antigen pulse at 37°C, followed by immediate washing off unbound antigen and incubation with gluten-specific T cell clones. Our data show that TG2-gluten complexes generated during this short incubation time are efficiently taken up by both DGP- and TG2-specific B cells and facilitated the proliferation of gluten-specific T cells (Fig 6).

## Discussion

Multivalent antigens are particularly effective at activating B cells due to their ability to crosslink B cell receptors on the cell surface [22–25]. Multivalency of B-cell antigens together with the provision of T-cell help is a credible mechanism for facilitating loss of B-cell tolerance to autoantigens in autoimmune diseases [22, 23]. In agreement with earlier results using transfected cell lines [11], we found that TG2-gluten multimers were superior to monomers in activating naive primary TG2-specific B cells and gluten-specific CD4^+^ T cells *in vitro*. We also found that such TG2-gluten complexes, even when merely a TG2 monomer, allow for multivalent presentation of gluten epitopes by DGP-specific B cells. TG2-gluten complexes can thus serve as powerful antigens for both DGP- and TG2-specific B cells and, when decorated with many different gluten epitopes, will allow both B cells to receive help from many different gluten-specific T cells. Sharing a common mode of activation could explain some of the striking resemblance in the repertoires of DGP- and TG2-specific plasma cells, such as restricted variable (V)-gene usage and limited number of somatic hypermutations [19, 21].

For the first time, we here document that pre-formed covalently crosslinked TG2-gluten complexes may elicit cognate interaction between TG2-specific B cells and gluten specific CD4^+^ T cells *in vivo*. In contrast to the in vitro assays, no significant differences between monomers and multimers was seen *in vivo* after intramuscular injection. Differences in stability or mobility to lymphoid inductive sites probably explain this discrepancy between *in vitro* and *in vivo* findings.

The key question remains whether such TG2-gluten complexes can also be generated *in vivo*, and where in the body active TG2 encounters gluten. Using our immunoglobulin KI model, we have previously demonstrated that there is no induction of B-cell tolerance to TG2, indicating that B cells are not exposed to TG2 during development nor in the periphery [12]. Priming of gluten-specific T cells and DGP- and TG2-specific B cells in celiac disease likely takes place in Peyer’s patches, organized lymphoid structures that play a central role in the induction of mucosal immune responses in the gut. Possibly TG2 derived from shed enterocytes reacts with gluten peptides in the gut lumen and forms TG2-gluten complexes that will be available to B cells in Peyer’s patches. Indeed, we have recent data showing that TG2-specific B cells incubated with a lysate of mouse enterocytes and native gluten peptides efficiently activated gluten-specific T cells and this was dependent on the presence of active TG2 [26]. No isopeptide-crosslinked complexes between TG2 and gluten peptides could be detected in that recent study, suggesting that thioester-bonded enzyme-substrate intermediate TG2-gluten complexes activated the TG2-specific B cells. Further research is needed to elucidate the true nature of the TG2-gluten interaction *in vivo*, and it seems apparent that future efforts must be focused on the gut.

## Supporting information

S1 Fig Adequate separation of TG2-gluten monomers and multimers as evaluated by SDS PAGE. Proteins were allowed to self-crosslink as previously described, then separated by size-exclusion chromatography before running on a gel. Leftmost lane: protein ladder, numbers indicate molecular weight. kD: kilodaltons. 1: TG2-α33merEEE control (not crosslinked/separated). 2: TG2-α33merEEE monomers. 3: TG2-α33merEEE multimers. 4: TG2 control (not crosslinked/separated). 5: TG2-α33mer monomers. 6: TG2-α33mer multimers.
(TIF)

S1 Raw images (PDF)
