# Supplementary figures and images for: TG2-gluten complexes as antigens for gluten-specific and transglutaminase-2 specific B cells in celiac disease
Source: PLoS One. 2021 Nov 3;16(11):e0259082. doi: 10.1371/journal.pone.0259082 (PMC8565743; doi:10.1371/journal.pone.0259082)

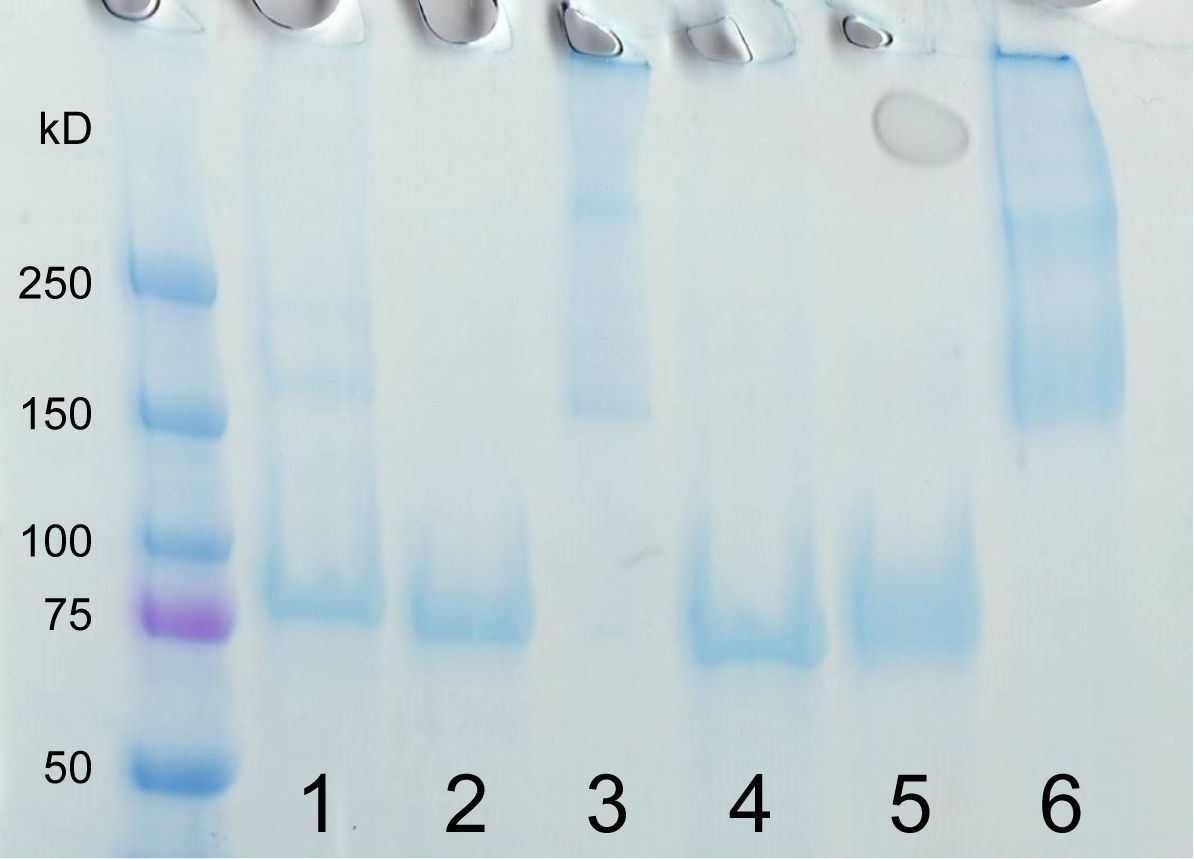

Supplement: S1 Fig — Proteins were allowed to self-crosslink as previously described, then separated by size-exclusion chromatography before running on a gel. Leftmost lane: protein ladder, numbers indicate molecular weight. kD: kilodaltons. 1: TG2-α33merEEE control (not crosslinked/separated). 2: TG2-α33merEEE monomers. 3: TG2-α33merEEE multimers. 4: TG2 control (not crosslinked/separated). 5: TG2-α33mer monomers. 6: TG2-α33mer multimers. (TIF) [file pone.0259082.s001.tif]
