# Supplementary material for: TG2-gluten complexes as antigens for gluten-specific and transglutaminase-2 specific B cells in celiac disease
Source: PLoS One. 2021 Nov 3;16(11):e0259082. doi: 10.1371/journal.pone.0259082 (PMC8565743; doi:10.1371/journal.pone.0259082)

Raw image file for Lindstad & Dewan et al.

Figure 5B

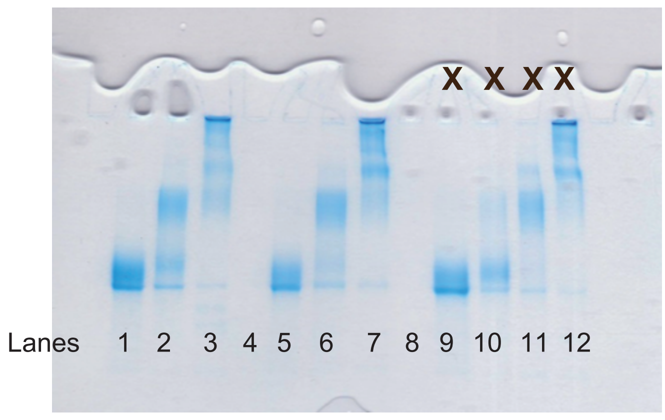

Ratio Rho-α33mer : FITC-ω34mer

1 2:1 monomers
2. 2:1 dimers, trimers
3: 2:1 multimers
4. empty
5. 17:1 monmers
6. 17:1 dimers, trimers
7. 17:1 multimers
8. empty
9. X
10. X
11. X
12. X

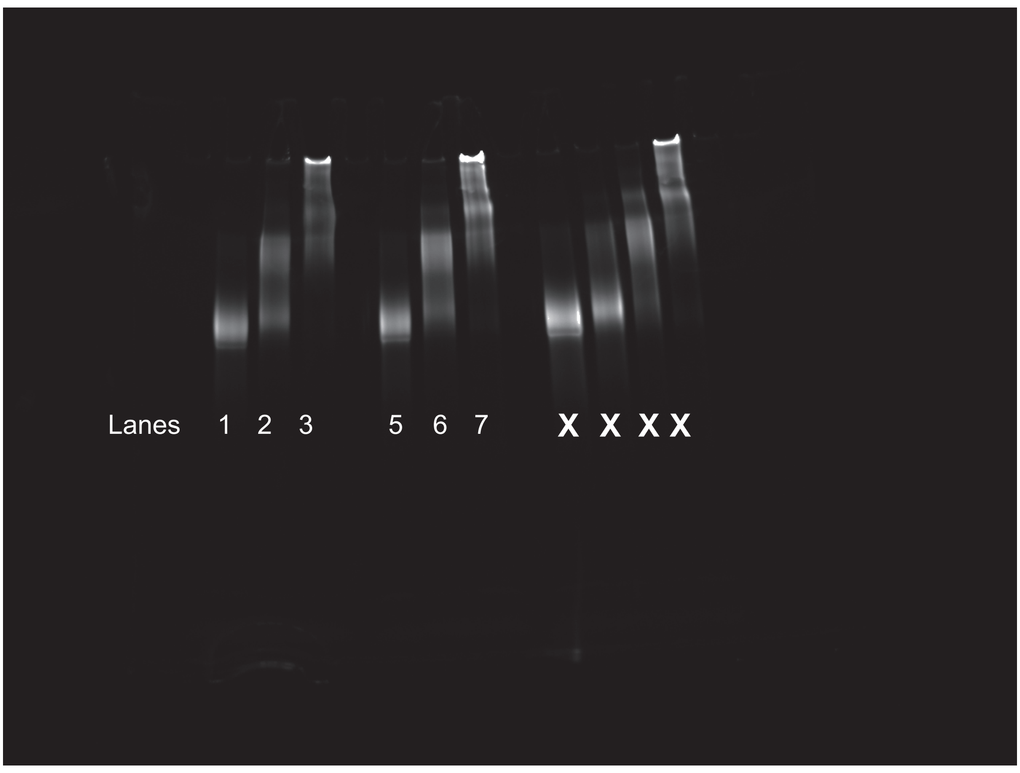

Scan was acquired using BioRad gel scanner with Rhodamine filter, picture is iverted for Fig. 5B.

No Mw ladder was used as it interferes with the Rhodamine scanning.

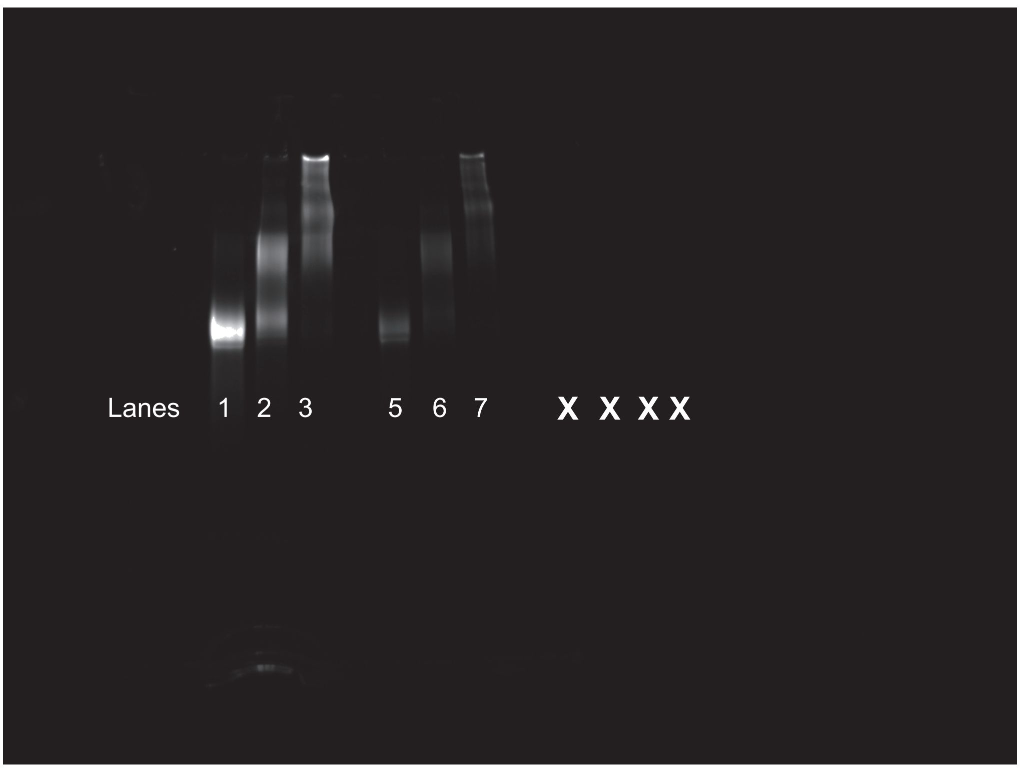

Scan was acquired using BioRad gel scanner with Fluorescein (FITC) filter, picture is inverted for Fig. 5B

Figure S1

Lanes 1 2 3 4 5 6

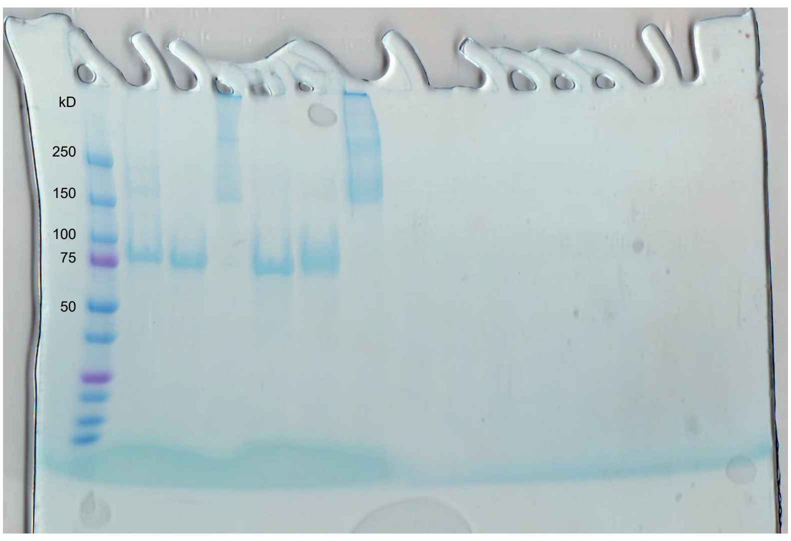

Supplement: S1 Raw images — (PDF) [file pone.0259082.s002.pdf]
